# Better Outcomes for Older people with Spinal Trouble (BOOST) trial: statistical analysis plan for a randomised controlled trial of a combined physical and psychological intervention for older adults with neurogenic claudication

**DOI:** 10.1186/s13063-020-04590-x

**Published:** 2020-07-21

**Authors:** Ioana R. Marian, Esther Williamson, Angela Garrett, Sarah E. Lamb, Susan J. Dutton

**Affiliations:** 1 Oxford Clinical Trials Research Unit, Centre for Statistics in Medicine, Nuffield Department of Orthopaedics, Rheumatology, and Musculoskeletal Sciences, Botnar Research Centre, University of Oxford, Old Road, Oxford, OX3 7LD UK; 2 Centre for Rehabilitation Research, Nuffield Department of Rheumatology, Orthopaedics and Musculoskeletal Sciences, University of Oxford, Oxford, UK; 3 College of Medicine and Health, Institute for Health Research, University of Exeter, St Luke’s Campus, Heavitree Road, Exeter, UK

**Keywords:** Statistical analysis plan, Randomised controlled trial, Neurogenic claudication, Lower back pain

## Abstract

**Background:**

Neurogenic claudication is a common spinal condition affecting older adults that has a major effect on mobility and implicitly independence. The effectiveness of conservative interventions in this population is not known. We describe the statistical analysis plan for the Better Outcomes for Older people with Spinal trouble (BOOST) randomised controlled trial.

**Methods/design:**

BOOST is a pragmatic, multicentre, parallel, two-arm, randomised controlled trial. Participants are community-dwelling adults, 65 years or older, with neurogenic claudication, registered prospectively, and randomised 2:1 (intervention to control) to the combined physical and psychological BOOST group physiotherapy programme or best practice advice. The primary outcome is the Oswestry Disability Index at 12 months. Secondary outcomes include the Short Physical Performance Battery, Swiss Spinal Stenosis Scale, 6 Minute Walk Test, Fear Avoidance Beliefs Questionnaire, and Tilburg Frailty Indicator. Outcomes are measured at 6 and 12 months by researchers blinded to treatment allocation. The primary statistical analysis is by intention to treat. Further study design details are published in the BOOST protocol.

**Discussion:**

The planned statistical analyses for the BOOST trial aim to reduce the risk of outcome reporting bias from prior data knowledge. Any changes or deviations from this statistical analysis plan will be described and justified in the final study report.

**Trial registration:**

This study has been registered in the International Standard Randomised Controlled Trial Number registry, reference number ISRCTN12698674. Registered on 10 November 2015.

## Background

Neurogenic claudication is a common condition affecting older adults [1]. The symptoms include discomfort and pain radiating from the spine into the buttocks and legs, weakness, gait changes, and fatigue [2]. Neurogenic claudication symptoms are thought to arise from pressure on nerves and blood vessels in the spinal canal due to a narrowing of spinal canal volume (lumber spinal stenosis). However, the relationship between radiological imaging results and symptoms is inconsistent across this population [2, 3].

Spinal surgery is a common treatment in people over 65 years of age, but is known to carry a risk of complications and is costly [4–6]. Before surgery, it is recommended that patients undergo conservative treatments such as physiotherapy. However, the effectiveness of physiotherapy in patients with neurogenic claudication is unknown [7].

BOOST is a randomised controlled trial aiming to generate evidence for non-surgical treatment of neurogenic claudication, for a physiotherapist-delivered combined physical and psychological intervention targeting age-associated changes in the musculoskeletal system and the psychological impact of pain. This paper reports the details of the analysis plan for the BOOST trial as agreed by the Data and Safety Monitoring Committee (DSMC) in July 2019 and has been prepared according to the published guidelines on the content of statistical analysis plans [8]. The trial is registered with the International Standard Randomised Controlled Trials database, reference number ISRCTN12698674.

## Methods and design

### Trial design

BOOST is a pragmatic, multicentre, parallel, two-arm, randomised controlled trial aiming to assess the clinical effectiveness of a physiotherapist-delivered, combined physical and psychological intervention for older adults with neurogenic claudication, compared with best practice advice.

Participants in the study are identified from National Health Service (NHS) physiotherapy and consultant spinal clinics and through a primary-care-based cohort study (the Oxford Pain, Activity, and Lifestyle (OPAL) study). Eligible participants are randomised 2:1 (intervention to control), stratified by study centre, gender (male, female), and age group (65–74 years and 75+ years), using variable block sizes of 3 and 6. Stratification by each factor helps to ensure any factor-related effect is equally distributed in the two trial arms. Participants are randomised after an eligibility assessment and informed consent has been given. The baseline assessment is completed at a research clinic appointment. Randomisation is performed via a secure, web-based randomisation system provided by the Oxford Clinical Trials Research Unit, consistent with UK Clinical Research Collaboration-approved standard operating procedures. This system ensures prospective registration and allocation concealment.

All study participants receive an initial 1-h appointment. During this time, participants randomised to the BOOST programme undergo an assessment and are prescribed an individualised exercise and walking programme in preparation for the group sessions. They then attend 12 group sessions delivered over 12 weeks. The physical component of the intervention is enhanced by a psychological programme consisting of education and discussion underpinned by cognitive behavioural techniques [9]. During and after the classes, participants undertake home exercises and receive two support telephone calls to encourage them to continue with the exercises. Participants randomised to the control arm (best practice advice) undergo an assessment and are provided with tailored advice and education during the initial 1-h appointment. Two further review sessions are permissible. Advice includes self-management strategies, home exercises, and encouragement to increase physical activity.

Physiotherapists delivering the interventions and participants cannot be blinded to treatment allocation. Research staff collecting follow-up data are blinded, and participants are asked not to share their treatment allocation with researchers. The trial statistician and research staff undertaking quality assurance checks are not blinded to treatment allocation. The remaining members of the trial management team, including those involved in data management, are masked to treatment allocation. A Data Management Plan which includes references to confidentiality, access, and security arrangements has been produced for the study. This is stored in the trial master file and is available on request from the BOOST study office. Full details of the trial design, study population, and study procedures have been published in the BOOST protocol [10].

### Objectives

The primary objective of the study is to estimate the clinical effectiveness of a physiotherapist-delivered intervention combining physical and psychological components for older adults with neurogenic claudication, compared with best practice advice, based on observed differences in the Oswestry Disability Index (ODI) [11] between the trial treatment groups 12 months after randomisation. The null hypothesis assumes that there is no difference in the ODI between the two treatment arms 12 months after randomisation.

Secondary objectives include exploring whether indicators of frailty, behavioural factors, and radiological (MRI) biomarkers can predict a response to physiotherapy treatment using pre-specified subgroup analyses.

#### Intervention details and process evaluation

Following randomisation, one group of participants are allocated to a group physiotherapy and psychology intervention and one group to best practice advice. By standardising delivery within and between sites and formal fidelity assessments of group delivery, quality assurance procedures are ensured [9].

The primary indicator of compliance in the group physiotherapy arm is the attendance of at least 9 (75%) of the 12 group sessions. Compliance with treatment will be reported separately for each treatment arm, with reasons why participants were unable to attend classes, where available. Fisher’s exact or chi-squared tests will be used to assess the association between compliance and treatment group.

Measures related to adherence to exercises are also collected using the Index of Habit [12] and two self-reported measures [13]. The *Index of Habit* consists of four questionnaire items measured on a 5-point Likert scale (1 = agree, 5 = disagree)*. Self-reported adherence to the home exercise programme* is assessed with one question on a 6-point Likert scale (1 = never, 6 = every day). *Self-reported satisfaction with own attempts to increase physical activity* is measured with a trial-specific question on a 5-point Likert scale (0 = very dissatisfied, 4 = very satisfied). In addition to group attendance, the effects of dose prescribed and received will be explored (including the level of effort as rated by the physiotherapist during the strength exercises).

Physical activity levels in the past week are measured using two items from the *Rapid Assessment Disuse Index* (“moving around the feet*”* and “time spent sitting”) [14]. The items can be scored from 1 (7 h or more per day) to 5 (less than 1 h per day).

*Self-efficacy recovery and maintenance* [15] questions relate to performing home exercises and help to understand maintenance of exercise and physical activity and participants’ confidence to restart their exercises after stopping. The questions are scored on a 4-point Likert scale (0 = not at all true, 3 = exactly true).

### Outcomes

#### Primary outcome

The primary outcome for this study is the ODI version 2.1a at 12 months, a widely used outcome measure for neurogenic claudication and in the field of back pain. It has high applicability due to its items on standing and walking [11, 16]. A between-group difference of 5 points and a baseline standard deviation of 15 are considered clinically significant. The ODI is a self-administered 10-item questionnaire designed to assess limitations in various activities of daily living. Participants are asked to consider their back and leg symptoms when answering the questionnaire, including discomfort, heaviness, aching, tingling, and numbness. Responses are not limited to the impact of back pain. We have excluded the item on participants’ sex lives, as this is most commonly unanswered. The remaining nine items are scored from 0 to 5, with 5 representing the highest disability. If a participant marks more than one statement in a question, the highest scoring (worst-case scenario) statement is selected as the true indicator of disability. The ODI index will be calculated by summing the scores, dividing by the total possible score, and multiplying by 100 to express the result as a percentage. The denominator will be reduced by 5 for every unanswered question. Percentages will be rounded to a whole number for convenience [11]. Total ODI scores will be analysed as continuous outcomes with a range of 0–100, where higher scores indicate greater disability. Score bandings may be used in descriptive analysis or to aid interpretation.

#### Secondary outcomes

Secondary outcomes include a range of clinical assessments and patient-reported outcome measures to evaluate the intervention’s impact on key treatment targets: neurogenic claudication symptoms, mobility, physical activity, strength, balance, frailty and falls, and cognitive and behavioural factors related to adherence to exercise and improving physical activity levels. Table 1 presents a summary of outcomes and when they are collected (baseline, 6 months after randomisation, and/or 12 months after randomisation). A more in depth description of the collected outcomes is presented below.

**Table 1:** Primary and secondary outcome measures and when they are collected

Outcome measures	Time points		
	Baseline	6 months	12 months
**Primary outcome**			
Oswestry Disability Index (V2.1a) [11, 16, 17]	√	√	√
**Secondary outcomes**			
***Clinical assessment***			
Sagittal alignment of the spine [18, 19]	√	√	√
6 Minute Walk Test [20]	√	√	√
Hand grip strength [21, 22]	√	√	√
Short Physical Performance Battery [23]	√	√	√
Standing balance	√	√	√
Walking speed	√	√	√
Chair stands	√	√	√
***Patient-reported outcomes***			
Fear avoidance beliefs questionnaire [24]	√	√	√
Swiss Spinal Stenosis Questionnaire [25]	√	√	√
Global rating of perceived change [26]	–	√	√
Satisfaction with treatment	–	√	√
Satisfaction with changes in back and leg problems	–	√	√
Satisfaction with attempts to increase physical activity	–	√	√
EQ-5D-5L [27, 28]	√	√	√
Change in mobility	√	√	√
Tilburg Frailty Indicator [29]	√	√	√
Self-report of falls and falls-related injury ProFANE [30]	√	√	√
Troublesomeness of back and leg problems [31]	√	√	√
Perceived ability to self-manage	√	√	√
Self-efficacy [15]	√	√	√
Modified gait self-efficacy	√	√	√
Self-efficacy recovery and maintenance	–	√	√
Index of Habit [12]	–	√	√
Self-report of adherence to home exercise programme	–	√	√
Physical activity – Rapid Assessment Disuse Index [14]	√	√	√

##### Clinical assessment

Postural alignment, the 6 Minute Walk Test, grip strength, and the Short Physical Performance Battery (SPPB) are measured during the research clinic assessment.

The degree of thoracic kyphosis is measured based on the *sagittal alignment of the spine, C7 to wall measure* [18, 19]. The participant removes their socks and shoes and stands in an upright position, with their back and sacrum against the wall and their hands by their sides. The researcher measures the distance in millimetres from the wall to the spinous process of the seventh cervical vertebrae using a ruler [10].

The *6 Minute Walk Test* [20] measures the distance that the participant is able to walk in 6 min. Before the test, the researcher asks the participant verbally if they have neurogenic claudication symptoms. If they do not have any symptoms when starting, the participant is asked to verbally indicate if they begin to experience symptoms during the test. The distance at which their symptoms begin is recorded by the researcher. Full test details are described in the protocol [10].

The researcher measures the participant’s *grip strength* using a Jamar® hand dynamometer and follows the protocol outlined by Roberts et al. [21, 22]. Three measurements are taken on each hand with at least 30 s rest between measurements on the same hand. The best of the six grip strength measurements is used as the summary measure. Full test details are presented in the protocol [10].

The *SPPB* [23] measures three aspects of physical performance: standing balance, walking speed, and the time taken to perform five chair stands as per details described in the protocol [10]. The three test sections are scored as follows:

1. *Standing balance*. Standing balance is rated on a scale of 0–4 according to the participant’s ability to maintain three test positions (side-by-side stance, semi-tandem, and full tandem) for 10 s.
2. *Walking speed.* Walking speed is measured twice (in seconds) and the better of the two times is used to score the test on a scale of 0–4.
3. *Chair stands.* The participant sits in a straight-backed chair with arms folded across their chest. They are asked to stand up straight five times in succession, as fast as they can. The time taken to perform the five chair stands (from the initial sitting position to the final standing position at the end of the fifth stand) is used to score the test on a scale of 0–4.

The overall SPPB score will be calculated by summing the scores for standing balance, walking speed, and chair stands. Participants unable to complete a test receive a section score of 0. The three section scores and the overall SPPB score (range 0 to 12) will be reported.

Baseline-specific assessments including patient reported measures of pain and other symptoms, mobility, and psychological factors, are conducted at the outset and will be described. We use the StarT Back Screening Questionnaire [32, 33], Nordic Pain Questionnaire [34], Attitude to Ageing Questionnaire - physical change subscale [35], self-reported current health conditions, change in mobility in the last year, use of walking aids, self-rated walking speed, Exercise Self-Efficacy (short version) [36], and intention to carry out home exercises [37].

The *StarT Back Screening Questionnaire* [32, 33] is an assessment for screening pain prognostic indicators evaluating physical and psychological factors. The tool includes nine items: back pain bothersomeness (1), leg pain (2), shoulder or neck pain (3), safety of physical activity (4), dressed more slowly (5), short distances walked (6), worrying thoughts (7), terrible back pain (8), and no enjoyment (9). All items are scored as positive (= 1). Items 2–9 are scored as positive if “agree” is marked and item 1 is scored as positive if participants mark “very much” or “extremely” bothered. The overall score (ranging from 0 to 9) is determined by summing all positive responses. Items 1, 4, 7, 8, and 9 form the psychosocial subscale (score ranging from 0 to 5). A psychological subscale score ≥ 4 classifies participants as high risk, an overall score ≥ 4 and psychological subscale score < 4 as medium risk, and an overall score < 4 as low risk.

The *Nordic Pain Questionnaire* [34, 38] adapted version is used to assess the presence of pain in various parts of the body, based on binary responses (yes or no) at baseline. We will use these responses to describe participants as having no pain, single-site pain, or multisite pain [39].

##### Participant-reported outcomes

Study participants are asked to complete a questionnaire at the research clinic visit or via post if they are not able to attend the clinic (follow-up only). Participant-reported outcomes collected, including pain and other symptoms, mobility and psychological factors, are described below.

The *Attitude to Ageing Questionnaire - Physical Change subscale* [35] is a self-reported measure for expressing attitudes towards the ageing process among older people. The Physical Change domain has eight items measured on a 5-point Likert scale (1 = strongly disagree, 5 = strongly agree). The total subscale score ranges from 8 to 40, with higher values indicating better outcomes.

Fear avoidance is measured using the *Fear Avoidance Beliefs Questionnaire - Physical Activity subscale* (FABQ PA) containing four items [24]. The total FABQ PA score is calculated by summing the scores, with the total ranging from 0 to 24 and higher values indicating a worse outcome.

The *Swiss Spinal Stenosis (SSS) Scale* [25, 40] is a condition-specific assessment developed for participants with lumbar spinal stenosis. The trial uses the SSS symptoms severity subscale containing seven items divided into two domains: pain (questions 1–4) and neuroischaemic (questions 5–7). Six questions are scored on a Likert scale from 1 to 5, with 1 indicating an absence of symptoms and 5 indicating very severe symptoms. One question on balance offers three answer options, scored 1, 3, or 5. The subscale score is calculated as the unweighted mean of all answered items provided no more than two responses are missing.

*The global rating of perceived change* [26] contains one question assessing the participants’ perceived change in back and leg pain symptoms over the past 6 months with answers on a 7-point Likert scale (0 = completely recovered, 6 = vastly worsened).

Participants’ *satisfaction with treatment*, *changes in back and leg problems*, and *increases in physical activity* are assessed in three questions following the format: “How satisfied are you with the exercises that you were given to help with your back and leg problems?” Answers to the questions are reported on a 5-point Likert scale (0 = very dissatisfied, 4 = very satisfied).

Health-related quality of life is measured using *the EuroQoL 5-Dimension 5-Level Scale* (EQ-5D-5L) [27]. The EQ-5D-5L can be used to report health-related quality of life in five dimensions. Each combination of answers can be converted into a health utility score where 1 represents perfect health, 0 indicates death, and negative values are possible [28]. It has good test-retest reliability and gives a single preference-based index value for health status that can be used for broader cost-effectiveness comparative purposes.

*Change in mobility* in the last 6 and 12 months is measured using a 5-point scale constructed for the trial. The questions follow the structure: “Compared with 1 year ago, how would you rate your walking in general? Much better now than 1 year ago; somewhat better than 1 year ago; about the same; somewhat worse than 1 year ago; or much worse now than 1 year ago” (1 = much better than 1 year ago, 5 = much worse than 1 year ago).

A range of measures are collected to capture constructs related to ageing. Frailty is measured using the *Tilburg Frailty Indicator* [29]. Scores range from 0 to 15, with higher values indicating more frailty. The score will be categorised into those above and below 5.

Information about falls and fall-related injuries is self-reported based on *Prevention of Falls Network Europe* (ProFANE) [30]. Participants state whether they have fallen once, more than once, or not at all in the past 6 months. They are asked to report if there were any fractures as a result of falling and the number of broken bones.

*Troublesomeness of back and leg problems* (including pain, aching, numbness, tingling, or heaviness) in the last 6 weeks is measured on a 6-point Likert scale question (0 = not at all troublesome, 5 = extremely troublesome) [31].

*Perceived ability to self-manage* is captured in one question (“We would like you to think about how you are managing your symptoms and your ability to walk and be mobile. How well do you feel that you are managing your back and leg problems today?”), reported on a 0 to 10 visual analogue scale (0 = not managing at all, 10 = managing extremely well).

Self-efficacy is measured during follow-up based on two perspectives: modified gait self-efficacy and self-efficacy recovery and maintenance [15, 41, 42]*.* Participants rate their confidence to walk half a mile on a single item from the 10-item *Modified Gait Self-efficacy Scale* (“How much confidence do you have that you would be able to safely walk a long distance such as 1/2 mile?”). The question score range is 0 to 10 (0 = no confidence, 10 = complete confidence). *Self-efficacy recovery and maintenance* is described in the intervention and process evaluation section.

The participant questionnaire also asks whether participants have been placed on a waiting list for a back or leg surgery in the 6 months before answering the question.

### Sample size

The sample size calculations required a minimum of 402 participants and a maximum of 540, finalised following a review of the sample size assumptions by the DSMC. No formal interim analysis of the primary outcome was performed. The calculations were based on the assumption that a between-group difference of 5 points in the ODI, with a baseline standard deviation of 15, is considered clinically significant. This is consistent with published estimates in older populations and those with neurogenic claudication [43, 44] and yields a standardised effect size of 0.33, which is a moderate effect size. At 80% power and 5% two-sided significance levels, the proposed sample size was 321 participants providing data at 12-month follow-up (214 in the intervention arm and 107 in the best practice advice arm). An inflation for potential loss to follow-up (20%) led to an overall target of 402 (268 intervention, 134 control).

At 90% power, a sample size of 429 (286 in the intervention arm and 143 in the control arm) was required and together with an inflation for potential loss to follow-up (20%) yielded an overall target of 540 (360 intervention, 180 best practice advice). The 20% loss to follow-up was based on recent experiences of rehabilitation trials with older participants [45].

Therapist effects are expected to be negligible based on data generated and published from a series of trials using similar standardised interventions. Our recent trials of hand exercises in rheumatoid arthritis and cognitive behavioural interventions for lower back pain generated an intracluster correlation of less than 0.0001. We anticipated that approximatively 20 therapists would deliver the intervention, treating an average of 12 to 15 participants each. A formal inflation for a therapist effect was not incorporated due to the generous loss to follow-up allowance, which should mitigate against any moderate to large therapist effects.

### Statistical analysis

#### General analysis principles

Data will be reported in accordance with the Consolidated Standards of Reporting Trials (CONSORT) guidelines for randomised controlled trials and the extensions for non-pharmacologic treatment interventions and patient-reported outcomes [46–48]. The distribution of variables, missing data, and outliers will be assessed in a blinded analysis of the data before the final data lock. The treatment code will be added to the database after the data has been cleaned. The primary analysis will be intention to treat (ITT): participants will be included in their randomised groups and effect estimates with their 95% confidence intervals (CIs) will be reported at a significance level of 0.05. Sensitivity analyses will examine the robustness of the primary analysis for the primary outcome in the population compliant with treatment using a complier average causal effect (CACE) analysis [49]. We have dealt with multiplicity in an accepted method by having a pre-specified primary end point and pre-specified analysis plan [48]. Analyses of secondary outcomes will be considered to be supportive of the primary outcome analysis, and conclusions of the trial will not be based on these outcomes. All analyses will be carried out using appropriate, validated statistical software such as STATA, R, or SAS. The version number used for the analysis will be reported.

#### Descriptive analyses

**Fig. 1:**
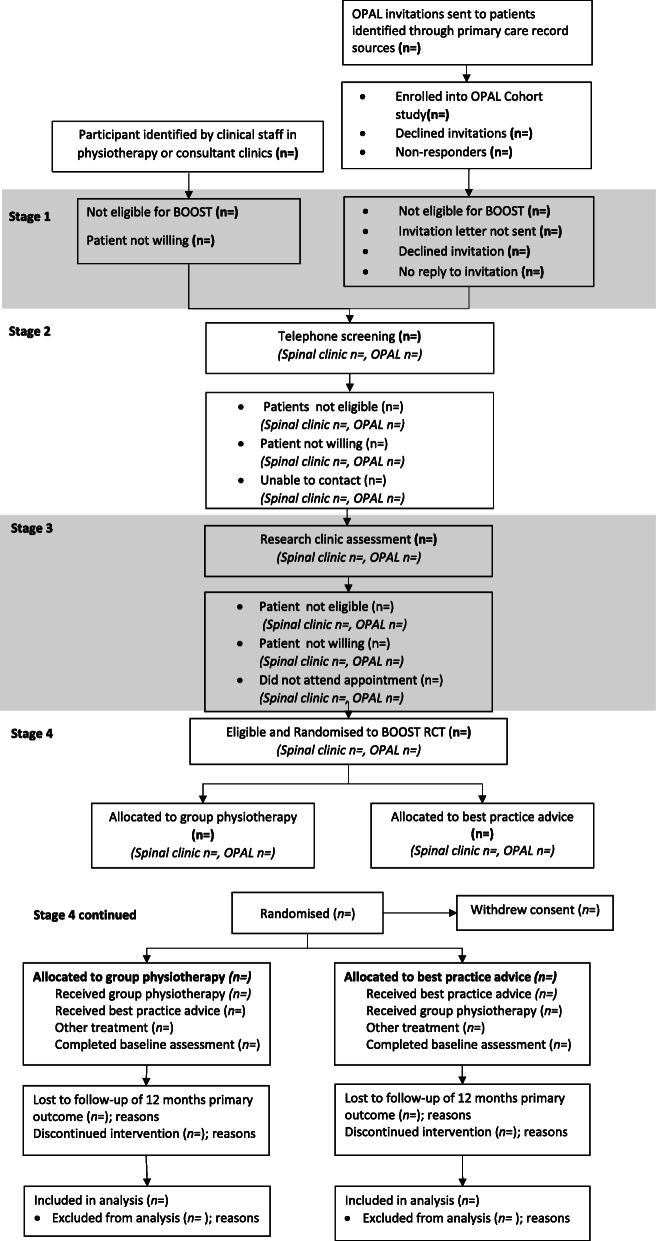
Recruitment flow chart of participants in the trial template

Participant flow through each stage of the trial will be summarised using a CONSORT flow chart, showing numbers of participants approached, eligible, ineligible by reason, consenting and randomised, receiving intended treatment, completing the study protocol, and analysed for the primary outcome (additional details listed in Fig. 1)*.* Participants who are randomised and retrospectively remove their consent to be in the study will be listed on the participant flow diagram, but no clinical data provided will be retained without their consent.

Participant baseline stratification factors (centre, age, and gender) and available demographics will be reported by treatment arm and reported as numbers with percentages for categorical variables and means and standard deviation or medians and interquartile range for continuous variables. No tests of statistical significance for differences between randomised groups in any baseline variable will be performed.

Baseline characteristics will be presented separately for the participants randomised, those included in the ITT primary outcome analysis, and those with no primary outcome follow-up. This will facilitate assessing whether outcomes are affected by attrition bias in each of the treatment arms [50]. The numbers (with percentages) of participants recruited via the OPAL cohort study and spinal clinics will be presented together with important baseline characteristics for these two recruitment sources. The age distribution by gender for the two intervention arms will also be explored.

The following demographic information is collected at baseline and will be presented:

- Gender
- Age
- Recruiting centre
- Recruitment source (OPAL or spinal clinic)
- Height; weight; body mass index
- Current alcohol and smoking behaviour
- Ethnicity
- Relationship status
- Employment
- Education
- Type of housing (past 6 months)
- Unpaid/paid carer
- Household income

#### Withdrawal from treatment and/or follow-up

The number (with percentage) of withdrawals from the trial by each study time-point and the numbers lost to follow-up for the primary outcome and reasons for withdrawal/loss will be reported. Associations between loss to follow-up, baseline characteristics, and treatment allocation will be explored. Any deaths (and their causes) will be reported separately. The number (with percentage) of withdrawals from treatment in the BOOST programme intervention arm before and after completing nine sessions and reasons for withdrawal will be reported.

#### Missing data

Data availability for the primary and secondary outcomes will be summarised from baseline to end of follow-up for the two treatment groups. The pattern of missing data (systematic or random) will be explored and the suitability of the missing at random (MAR) assumption (i.e. the likelihood that data are missing does not depend on the values of the missing data [51]) considered. Reasons for missing data will be presented where known.

Missing items within scales will be dealt with based on published instrument recommendations where available. Missing primary and secondary follow-up outcomes will be handled as part of the likelihood-based estimation of the mixed effects model in the primary analysis, assuming the MAR assumption holds [52].

#### Analysis of primary outcome

The primary outcome—ODI 12 months after randomisation—will be analysed and reported for each of the two treatment groups in the ITT population. The difference in ODI between the two treatment arms will be estimated using a linear mixed-effects regression multilevel model. The model will include random effects to account for any heterogeneity in response due to the recruitment centre and observations within-participant, and fixed effects for participant age, gender, and baseline ODI. The distribution of treating therapists across each centre will be assessed. It is anticipated that any therapist effects will be accounted for as part of the random effect for centre in the mixed-effects model. However, the exploration of therapist effects may be complicated because each participant may be treated by more than one therapist. This will be explored during the sensitivity analyses.

The primary outcome analysis will be conducted using the available data and missing follow-up ODI outcomes will be handled as part of the likelihood-based estimation of the mixed effects model in the primary analysis, assuming the MAR assumption holds [52]. Statistical tests will be two-sided and considered to provide evidence for a significant difference if *p* values to three decimal places are less than 0.05 (5% significance level). The adjusted estimate effects and associated 95% CI will be reported. Preliminary checks will assess the linear regression assumptions of normality and homoscedasticity of residuals. If severe departure from normality is identified, the first approach will be data transformation or the use of a different metric such as change from baseline to attain normality. If the data cannot be transformed to reflect normality, then the Mann-Whitney *U* test will be used (in this case, no further adjustments will be made) and the medians and interquartile ranges will be reported for each treatment arm. The ODI index will be calculated and reported at baseline and at 6 and 12 months, with the correlation between baseline and these follow-up time points. Secondary outcomes will provide supporting evidence.

A secondary CACE analysis using the same statistical model described for the primary analysis will be conducted for the primary outcome ODI in the population compliant with the intervention as defined in the *Compliance* section [49]. This analysis will be based on the exclusion restriction assumption, i.e. that members of the best practice advice group have the same probability of noncompliance as the group physiotherapy members and that simply offering non-compliers their randomised treatment has no effect on the outcome [53, 54].

#### Analysis of secondary outcomes

All secondary analyses will be conducted following similar methods to the primary outcome analysis, using linear regression for continuous outcomes and logistic/multinomial logistic regression for categorical outcomes, depending on the outcome measure. The models used to estimate the treatment effect will adjust for similar variables as used in the primary analysis.

The number of fractures following a fall will be collected at 6 and 12 months follow-up and reported as numbers of people with no fractures, one fracture, and more than one fracture. The association with treatment arm will be assessed using a Fisher’s exact or chi-squared test and will be reported with their associated 95% CIs and *p* values.

#### Safety

The number of adverse events and serious adverse events occurring while a participant is in the study and their relatedness will be reported. The number of participants experiencing serious adverse events in the two treatment groups will be compared by examining the 95% CIs for the difference in incidence. The analysis will be conducted for the ITT population.

#### Pre-specified subgroup analysis

Subgroup effects for pre-specified subgroups will be analysed and explored for the primary outcome ODI using interaction with treatment tests and displayed using forest plots [55]. The analysis will use primary outcome analysis model and will include an additional subgroup-by-treatment interaction term for each subgroup separately. Any formal subgroup testing will use a statistical significance level of 0.01; however, subgroup analyses are considered as exploratory and hypothesis generating. The following subgroups will be analysed (based on baseline characteristics):

- Age (65–74 years/75 years+)
- Gender (male/female)
- Tilburg Frailty Index scores (0–4/5) [29]
- Fear Avoidance Beliefs Questionnaire scores (0–14/15+) [56]
- STarT Back Screening Risk Stratification score (low-risk/medium-risk/high-risk groups) [32]
- Hand grip strength (men: < 30/30+; women: < 20/20) [57]
- Short Physical Performance Battery Scores (SPPB 0–6 low performance/SPPB 7–9 intermediate performance/SPPB 10–12 high performance) [57].

Additional pre-specified subgroups defined by MRI scan parameters will be used to predict change in ODI scores between baseline and 12 months. These subgroups are defined as follows:

- Central canal stenosis, defined by minimum dural sac cross-sectional area < 100 mm^2^ (present/absent)
- Lateral recess stenosis, defined by minimum lateral recess depth < 3 mm (present/absent)
- Foraminal stenosis, defined by minimum diameter < 3 mm (present/absent)
- Single-level vs multi-level central stenosis, defined by dural sac cross-sectional area ≤ 100 mm^2^ and the number of central stenosis levels (no spinal level with DS-CSA ≤ 100 mm^2^/a single DS-CSA ≤ 100 mm^2^/more than one level with DS-CSA ≤ 100 mm^2^)
- Qualitative grading of central canal stenosis, defined by degree of stenosis based on the amount of cerebrospinal fluid space around the nerve roots of the cauda equina [58] (grades A and B/grades C and D)
- Qualitative grading of nerve root entrapment in the lateral recess, defined by the size of the lateral recess and entrapment of the transiting nerve root within the lateral recess. Higher grades indicate more severe stenosis (no entrapment (grades 0 and 1)/entrapment (grades 2 and 3)) [59]
- Qualitative grading of nerve root entrapment in the neural exit foramen defined by degree of nerve root entrapment within the neural exit foramen. High grades indicate more severe compression (no foraminal nerve root entrapment (grade 0)/foraminal nerve root entrapment (grade 1, 2, or 3) [60]

#### Sensitivity analyses

Sensitivity analyses to confirm the robustness of the ITT primary outcome analysis conclusions will be conducted using different analysis approaches.

##### Therapist effects

Where possible, the same physiotherapists will treat each group of participants throughout the treatment period. The primary analysis model described assumes that any therapist effects are incorporated into the random effects for each centre. Sensitivity analysis will further explore the potential impact of clustering due to therapist effects and the possibility of a participant being treated by more than one physiotherapist. The data will be explored and the most appropriate method selected. For example, if in most or all cases one therapist has provided the majority of treatment for a patient, then this therapist will be selected for the analysis or standard error estimates that are robust against clustering will be included in the mixed effects model used in the primary analysis.

##### Missing data assumption analysis (ITT population)

If data are missing, we will conduct sensitivity analyses to assess the robustness of the primary trial results in light of the assumptions made about the underlying missing data mechanism. Most analyses assume data to be MAR or missing completely at random. The sensitivity analysis will therefore assume missing not at random: missing outcomes will be assumed to be worse or better than the observed outcomes [61]. The *rctmiss* command in Stata may be used for such sensitivity analysis.

### Supplementary analyses and outcomes

#### Mediation analysis

We will conduct mediation analyses to evaluate treatment mechanisms and exploratory analyses of exercise dose effects, including profiling treatment-response trajectories. These analyses are detailed in a separate statistical analysis plan.

### Health economics

All cost effectiveness analyses will be undertaken by the health economist following a separate health economics analysis plan written by the trial health economist.

## Discussion

The BOOST trial will provide data on the effects of the BOOST physiotherapy intervention on the ODI in older adults with neurogenic claudication 12 months after randomisation, compared with best practice advice. One study limitation is that we cannot mask participants to their treatment allocation, as the type of intervention received is clearly notable.

This paper describes the planned statistical analyses for BOOST. Any changes from the protocol or statistical analysis plan will be described and justified in the final statistical report. The aim of pre-specifying the analysis is to reduce the risk of data-driven results and outcome reporting bias [16].

## Trial status

Recruitment into the trial opened on 1 August 2016 and closed on 31 August 2018. We have recruited 438 participants from 14 sites. There were 14 trial sites in the following areas: Oxford, Cambridgeshire, Gloucestershire, Birmingham (3 sites), Yorkshire (2 sites), Croydon, Dorset, Wiltshire, Cheshire, Merseyside, Liverpool, and Wirral. Follow-up for the trial outcome data is ongoing and expected to be completed by October 2019. Analysis will begin once this follow-up is complete.

## Data Availability

The datasets analysed during the current study will be available from the corresponding author on reasonable request.

## Abbreviations

BOOST: Better Outcomes for Older People with Spinal Trouble
CACE: Complier average causal effect
CI: Confidence interval
CONSORT: Consolidated Standards of Reporting Trials
DSMC: Data and safety monitoring committee
EQ-5D-5L: EuroQoL 5 Dimension 5 Level Scale
FABQ PA: Fear Avoidance Belief Questionnaire Physical Assessment
ITT: Intention to treat
MAR: Missing at random
MRI: Magnetic resonance imaging
NHS: National Health Service
NIHR: National Institute for Health Research
ODI: Oswestry Disability Index
OPAL: Oxford Pain, Activity and Lifestyle
SPPB: Short Physical Performance Battery
SSS: Swiss spinal stenosis
UK: United Kingdom
